# Predictors of the Use of a Mental Health–Focused eHealth System in Patients With Breast and Prostate Cancer: Bayesian Structural Equation Modeling Analysis of a Prospective Study

**DOI:** 10.2196/49775

**Published:** 2023-09-12

**Authors:** Nuhamin Gebrewold Petros, Jesper Alvarsson-Hjort, Gergö Hadlaczky, Danuta Wasserman, Manuel Ottaviano, Sergio Gonzalez-Martinez, Sara Carletto, Enzo Pasquale Scilingo, Gaetano Valenza, Vladimir Carli

**Affiliations:** 1 National Centre for Suicide Research and Prevention of Mental Ill-Health Department of Learning, Informatics, Management and Ethics Karolinska Institute Stockholm Sweden; 2 National Centre for Suicide Research and Prevention of Mental Ill-Health Centre for Health Economics, Informatics, and Health Services Research Stockholm Health Care Services Stockholm Sweden; 3 Stockholm Centre for Health and Social Change Department of Psychology, School of Social Sciences Södertörn University Stockholm Sweden; 4 Life Supporting Technologies Universidad Politécnica de Madrid Madrid Spain; 5 Department of Clinical and Biological Sciences University of Turin Turin Italy; 6 Research Center "E.Piaggio" School of Engineering University of Pisa Pisa Italy

**Keywords:** mental health, eHealth system, perceived usefulness, structural equation modeling, cancer, NEVERMIND system, usability, digital health, Technology Acceptance Model

## Abstract

**Background:**

eHealth systems have been increasingly used to manage depressive symptoms in patients with somatic illnesses. However, understanding the factors that drive their use, particularly among patients with breast and prostate cancer, remains a critical area of research.

**Objective:**

This study aimed to determine the factors influencing use of the NEVERMIND eHealth system among patients with breast and prostate cancer over 12 weeks, with a focus on the Technology Acceptance Model.

**Methods:**

Data from the NEVERMIND trial, which included 129 patients with breast and prostate cancer, were retrieved. At baseline, participants completed questionnaires detailing demographic data and measuring depressive and stress symptoms using the Beck Depression Inventory–II and the Depression, Anxiety, and Stress Scale–21, respectively. Over a 12-week period, patients engaged with the NEVERMIND system, with follow-up questionnaires administered at 4 weeks and after 12 weeks assessing the system’s perceived ease of use and usefulness. Use log data were collected at the 2- and 12-week marks. The relationships among sex, education, baseline depressive and stress symptoms, perceived ease of use, perceived usefulness (PU), and system use at various stages were examined using Bayesian structural equation modeling in a path analysis, a technique that differs from traditional frequentist methods.

**Results:**

The path analysis was conducted among 100 patients with breast and prostate cancer, with 66% (n=66) being female and 81% (n=81) having a college education. Patients reported good mental health scores, with low levels of depression and stress at baseline. System use was approximately 6 days in the initial 2 weeks and 45 days over the 12-week study period. The results revealed that PU was the strongest predictor of system use at 12 weeks (β_use at 12 weeks is predicted by PU at 12 weeks_=.384), whereas system use at 2 weeks moderately predicted system use at 12 weeks (β_use at 12 weeks is predicted by use at 2 weeks_=.239). Notably, there were uncertain associations between baseline variables (education, sex, and mental health symptoms) and system use at 2 weeks, indicating a need for better predictors for early system use.

**Conclusions:**

This study underscores the importance of PU and early engagement in patient engagement with eHealth systems such as NEVERMIND. This suggests that, in general eHealth implementations, caregivers should educate patients about the benefits and functionalities of such systems, thus enhancing their understanding of potential health impacts. Concentrating resources on promoting early engagement is also essential given its influence on sustained use. Further research is necessary to clarify the remaining uncertainties, enabling us to refine our strategies and maximize the benefits of eHealth systems in health care settings.

## Introduction

### Background

Technological advancements have led to the emergence of eHealth systems as potential tools to enhance the delivery of health care services. The concept of eHealth systems refers to health services and information delivered or enhanced through the internet and related technologies. These self-management tools provide patients with the ability and skills to improve their health by self-monitoring and receiving personalized feedback [1,2]. An area in which eHealth tools have shown promise is the treatment of depression, a prevalent comorbidity in patients with cancer [3,4].

Patients with breast and prostate cancer in particular face unique challenges associated with their diagnoses, such as body image concerns, sexual dysfunction, and hormonal imbalances, which can contribute to an increased risk of developing depression and stress and significantly affect an individual’s well-being and daily functioning [5,6]. Over the past 2 decades, a growing body of research has demonstrated the efficacy of eHealth interventions for the treatment of depression and stress [7-9].

However, the adoption and use of eHealth interventions for depression treatment in patients with cancer remains suboptimal. This is due to several factors, including limited awareness of eHealth interventions’ effectiveness, complex user interfaces or designs, and a lack of integration into health care systems, which necessitates a better understanding of the factors that drive their use [10,11]. As such, the role of usability and acceptability becomes an essential focal point in the use of eHealth interventions, with adequate attention paid to what influences the ease of use and acceptance by patients.

### Prior Work and Theoretical Frameworks

Research highlights the importance of considering user-centered design and user experience, such as user engagement and user satisfaction, to ensure accessibility and effectiveness for a wide range of users, including those with mental health issues [2]. Similarly, a recent pilot study by Chow et al [12] identified the need to improve the usefulness and satisfaction of mental health apps in patients with breast cancer to increase user engagement. Worse mental conditions such as high depressive and stress symptoms also pose challenges such as reduced motivation and engagement and skepticism about digital interventions [13]. Similarly, a study by Lally et al [14] also found that the total time users spent on the CaringGuidance program—an autonomous web-based platform providing psychoeducation and facilitating self-management of distress—after a breast cancer diagnosis, the number of log-ins, and the number of program components viewed did not correlate with distress levels. Instead, the depth of engagement and the users’ ability to find the support they need from the program appear to be the more crucial factors.

**Figure 1:**
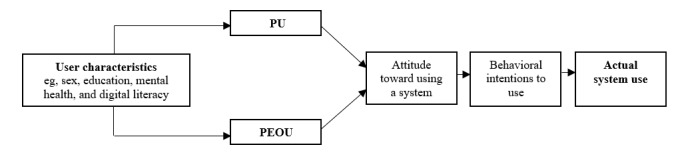
The Technology Acceptance Model (adopted and modified from Davis [15]). PEOU: perceived ease of use; PU: perceived usefulness.

A common and relatively easy-to-understand theoretical framework to comprehend and investigate user acceptance of new technologies is the Technology Acceptance Model (TAM), focusing on perceived ease of use (PEOU) and perceived usefulness (PU) [15]. According to the TAM, an individual’s likelihood of adopting and using technology is influenced by their perception of its ease of use and usefulness in achieving desired outcomes. For patients already grappling with health challenges, any perceived complexity or lack of immediate value can severely limit their engagement with eHealth solutions. Although the TAM has been validated empirically, incorporating more external user characteristics such as age, socioeconomic status, and mental health factors (eg, depression and stress) can improve the specificity and exploratory utility of this model (Figure 1).The TAM is an apt model for our study, which seeks to understand the adoption and use of the NEVERMIND system among patients with breast and prostate cancer with varying levels of depressive and stress symptoms.

### The NEVERMIND System

The NEVERMIND system was developed to reduce depressive symptoms in patients diagnosed with 5 somatic illnesses. The system comprises a mobile app and a sensorized T-shirt. The T-shirt collects physiological data, whereas the app gathers mental health questionnaires, both working together to predict depressive symptom levels. The system facilitates self-management of mental health symptoms in patients with somatic illnesses by allowing them to monitor their mental health and providing personalized feedback [16]. The effectiveness of the NEVERMIND system has been demonstrated in a randomized controlled trial (RCT) [9], and its acceptability and usability have been evaluated through usability questionnaires, with a higher favorability of the mobile app among female individuals and a higher use among male individuals [17].

### Goal of This Study

Although previous studies have provided valuable insights into the factors influencing the adoption and use of eHealth systems in general, few have specifically explored the role of baseline mental health symptoms, early engagement, PEOU, and PU within the context of the TAM, particularly in patients with breast and prostate cancer. In addition, most of the existing literature relies on traditional frequentist methods, which cannot fully investigate the relationship between theory and data collected from the system.

To address these gaps, our study uses Bayesian structural equation modeling (SEM), or more specifically, a path analysis, also called structural regression. This method offers several advantages over traditional frequentist methods. Bayesian methods allow for the incorporation of knowledge from previous research, enhancing the robustness and reliability of the drawn inferences. Moreover, Bayesian SEM excels in handling complex modeling assumptions more effectively than classic SEM, which typically uses maximum likelihood estimation [18]. These assumptions include the ability to manage complex distributions and nonlinear relationships and tackle challenges such as nonnormality, interactions, and measurement errors. This comprehensive approach enables a more nuanced interpretation of the interplay among variables.

This study, based on the TAM, aimed to explore the relationships among sex, education, baseline depressive and stress symptoms, initial use, PEOU, PU, and the use of the NEVERMIND eHealth system among patients with breast and prostate cancer.

To investigate the uncertainties in predicting the actual use of the NEVERMIND eHealth system within the TAM, the following hypotheses were formulated:

1. Male individuals, individuals with a higher educational level, and those exhibiting more depressive and stress symptoms are likely to use the system at 2 weeks.
2. Higher system use at 2 weeks is likely to lead to a higher PEOU at 4 weeks.
3. Higher system use at 2 weeks will lead to higher system use at 12 weeks.
4. Higher PEOU at 4 weeks will lead to a higher PEOU at 12 weeks.
5. Higher PEOU at 12 weeks will lead to higher system use at 12 weeks.
6. Higher PU at 12 weeks will lead to higher system use at 12 weeks.

**Figure 2:**
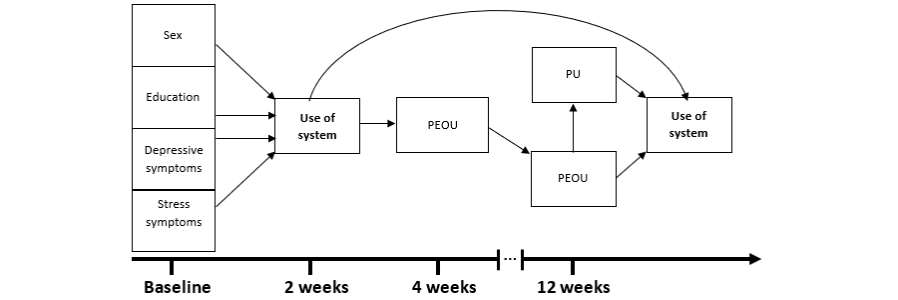
Model of the study. PEOU: perceived ease of use; PU: perceived usefulness.

These hypotheses are summarized in the study’s model (Figure 2). The model incorporates the TAM, but some components (attitudes toward using the system and behavioral intentions to use the system) were not measured in the main study; thus, they were not included in the model of this study.

## Methods

### Study Design

This study used a longitudinal design to explore the relationships among sex, educational level, baseline depressive and stress symptoms, PEOU, PU, and the use of the NEVERMIND eHealth system among patients with breast and prostate cancer. Participants were recruited from 2 large oncology centers, one specializing in breast cancer and the other in prostate cancer, in Pisa and Turin, Italy. Comprehensive details regarding the design, content, and functionality of the NEVERMIND system have been described in previous publications [9,17] (German Clinical Trials Register RKS00013391).

### Recruitment

#### Overview

Patients with prostate cancer were at an advanced stage (stage IV) at the time of recruitment. All treatments, with the exception of adjuvant androgen deprivation therapy, had been completed at least a month before their inclusion in the study. Similarly, patients with breast cancer were at stage III or IV at the time of recruitment. All treatments, barring hormonal or trastuzumab therapy, had been completed at least one month before their participation in the study. More extended inclusion and exclusion criteria for the NEVERMIND RCT have been described in detail in the protocol of the study [16]. As this is a secondary data analysis, the inclusion and exclusion criteria only refer to the subsample for this study.

#### Inclusion

Eligible participants were patients diagnosed with either breast or prostate cancer who were part of the NEVERMIND RCT study who were allocated to the NEVERMIND eHealth system.

#### Exclusion

Patients with breast and prostate cancer allocated to treatment as usual were excluded. Patients who belonged to the NEVERMIND intervention group but who dropped out of the study before receiving the NEVERMIND system were also excluded. Patients were also excluded if they had missing data on any of the variables of interest.

### Data Collection

#### Overview

**Figure 3:**
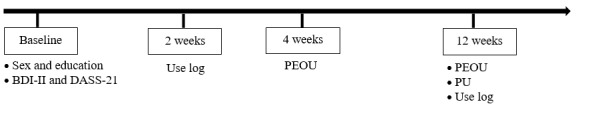
Timeline of data collection. BDI-II: Beck Depression Inventory–II; DASS-21: Depression, Anxiety, and Stress Scale–21; PEOU: perceived ease of use; PU: perceived usefulness.

Participants were asked to complete a set of questionnaires at baseline assessing their demographic information, depressive symptoms, and stress symptoms. Following the completion of the baseline questionnaires, participants were introduced to the NEVERMIND eHealth system and given a brief overview of its use. They were instructed to use the system for a period of 12 weeks, engaging daily with the app and at least twice a week with the sensorized T-shirt. Participants completed an interim follow-up questionnaire at 4 weeks and another questionnaire after the 12-week use period. The questionnaire included items assessing PEOU and PU using validated scales adapted to the eHealth context. The timeline of data collection is summarized in Figure 3.

A description of each variable is provided in the following sections.

#### Demographic Variables

Baseline sociodemographic data were collected for all patients recruited to the study. These data included sex and educational level. Educational level was dichotomized into low (below college or diploma) and high (college or above).

#### Mental Health Variables

Depressive symptoms were measured using the Beck Depression Inventory–II (BDI-II) [19], and stress symptoms were measured using the Depression, Anxiety, and Stress Scale (DASS-21) [20]. The BDI-II is a widely used 21-item self-report inventory that measures the severity of depressive symptoms in adults and adolescents, with each item rated on a scale from 0 to 3 based on the intensity of the symptom. The BDI-II score is calculated by adding the scores of its 21 items, with total scores ranging from 0 to 63, where higher scores signify more severe depressive symptoms. The Stress Scale of the DASS-21 is a 7-item subscale that assesses the respondent’s experience of stress symptoms over the past week. Each item is rated from 0 (*did not apply to me at all over the last week*) to 3 (*applied to me very much or most of the time over the last week*). The total is then doubled to align with the full version of the DASS-21, leading to a possible score range from 0 to 42, with higher scores indicating higher levels of stress.

#### Use of System

Patients in the intervention group were provided with the NEVERMIND system, which they were instructed to use for a period of 12 weeks. The system automatically collected data on each patient’s use of the mobile app and sensorized shirt without relying on patient self-reports. Each module of the mobile app recorded use data by distinct days of use and log data, which reflected instances in which a patient opened the app but did not necessarily engage with it or the modules or send any data to the server. Similarly, the sensorized shirt, via a docking station, transmitted use data to a remote server. These data were also recorded in terms of distinct days of use and log data. We computed 2 use variables for analysis, the first reflecting system use in the initial 15 days (2 weeks) and the second variable representing use over the entire 12-week study period.

#### PEOU Questionnaire

A questionnaire about the PEOU was administered to patients after 4 weeks of use and again after using the system for 12 weeks. PEOU is a measure of acceptability and is defined as “the degree to which a person believes that using a particular system would be free of effort” [15]. The PEOU questionnaire was developed by the Polytechnic University of Madrid according to the TAM. The questionnaire is a 9-item Likert scale ranging from 1 (*very difficult*) to 5 (*very easy*). Patients rated, for example, how easy it was to report and manage diet goals. The questionnaire was used as a continuous scale. The full questionnaire can be found in Multimedia Appendix 1.

#### PU Questionnaire

PU is defined as the “subjective perception of users regarding how much using a certain technology will improve the performance of their work” [15]. The questionnaire is a 10-item Likert scale that was developed by the Polytechnic University of Madrid according to the TAM. The questionnaire includes 10 positively worded statements, and patients were asked to rate their agreement with the statements on a scale from 1 (*strongly disagree*) to 5 (*strongly agree*). The full questionnaire can be found in Multimedia Appendix 2. The questionnaire was used as a continuous scale.

### Power

This study comprised a secondary data analysis using the data set from the primary NEVERMIND trial. For this analysis, we focused only on patients with breast and prostate cancer who were part of the intervention group, which consisted of 129 participants.

The sample size required for SEM analysis depends on various factors, such as the number of variables, the anticipated effect size, and the complexity of the model. For SEM, a rule of thumb is to have 10 to 20 cases per estimated parameter [21]. In the proposed model, we had 9 variables: baseline depressive and stress symptoms, sex, educational level, use at 2 weeks, PEOU at 4 and 12 weeks, PU at 12 weeks, and use at 12 weeks. On the basis of this recommendation, the sample size should be 90 to 180.

A total of 752 patients with breast cancer were approached to be included in the study. Of these 752 patients, 448 (59.6%) met the inclusion criteria. Of the 448 patients, 255 (56.9%) agreed to participate. These participants were then randomized, with 129 patients assigned to the NEVERMIND intervention group. In the intervention group, 83.7% (108/129) of patients completed the study, whereas 16.3% (21/129) of patients dropped out after completing the baseline questionnaires but before receiving the NEVERMIND system. Taken together, as patients were excluded if they had missing data on any of the variables of interest, the total sample size that we conducted the analysis on was 100. Although our sample size of 100 should be adequate to detect medium effects, it is worth noting that the power of SEM analyses can also be influenced by other factors, such as the nonnormality of data, missing data, and model misspecification [22].

### Statistical Analysis

#### Overview

Descriptive statistics were calculated for all variables, including participants’ demographic characteristics, baseline depressive and stress symptoms, and use patterns of the NEVERMIND system. Bayesian SEM was used as the statistical technique to analyze the relationships among the different parameters. Bayesian SEM was chosen for this specific research question for several reasons: (1) Bayesian SEM may be more robust with small sample sizes compared with traditional frequentist methods as it allows for the incorporation of previous information about model parameters, which can improve the precision of the estimates and produce reasonable results with small to moderate sample sizes [23]; (2) Bayesian SEM can estimate complex models with multiple parameters that might be too intricate for frequentist methodologies such as maximum likelihood [18], which is particularly relevant when examining the interrelationships among a large number of variables within a path analysis framework; and (3) Bayesian SEM enables us to incorporate previous knowledge of model parameters from previous research, which can enhance accuracy while estimating the posterior distribution for the model parameters [24].

In this study, a path analysis through Bayesian SEM was used to estimate relationships among different constructs simultaneously while accounting for previous information about the model parameters and estimating posterior distributions for these parameters based on the observed data. The steps outlined in the following sections were undertaken to set up the analysis for the Bayesian SEM.

#### Selection of Priors

The precision of Bayesian methods depends on accurate and informative prior distributions. Noninformative or default software settings can cause inaccurate estimates that are worse than frequentist estimates [18]. Consequently, choosing priors should incorporate previous beliefs gathered from relevant studies and meta-analyses or expert opinions. Prior distributions for the model parameters were chosen based on three different sources, in order of prioritization: (1) previous research, (2) weakly informed priors elicited from the authors of the study (ie, expert opinion), and (3) default prior from the *Blavaan* package in R (R Foundation for Statistical Computing) modified in the prior convergence analysis to avoid divergencies in the model to allow the model to run based on prior assumptions. Thus, expert opinions and default priors were only used when no previous empirical findings were available.

To identify effect sizes from previous research, a search was conducted on August 26, 2022, on PubMed. The search was broad enough to make sure that any relevant studies were reviewed. The search included the words “user characteristics” AND “usability” OR “usage” and “eHealth.”

#### Conversion and Aggregation of Effect Sizes

The effect size data obtained from previous research were in different formats, such as odds ratios for categorical variables and regression coefficients for continuous variables. All effect sizes were converted and aggregated to means and SDs for regression coefficients to be compatible with the input requirements of the *Blavaan* package in R. Odds ratios were recalculated into correlation coefficients in 2 ways. In case contingency table data were available, the φ coefficient was computed using the *ci.phi* function in R as it considers differences in group size. If only odds ratios were available, the effect size package in R with the function *oddsratio_to_r* was used. Data were aggregated when multiple studies reported the same effect and used the same methodology, as in a meta-analysis. The *meta* package in R, along with the *metacor* function, was used for this purpose. The aggregated value of the common effects was then used as the aggregate measure. When a single article reported an effect size, it was used as a prior for that specific pair of variables.

#### Conversion and Aggregation of SDs

Priors for the SDs were not reported for any of the effects. Thus, these were computed using the following formula:

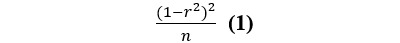



where *r* is the correlation coefficient and *n* is the number of variables, which was 2 for all cases [25]. In cases in which several articles reported effect sizes, aggregation of the SDs was performed by converting them to variances and weighting each variance by the number of participants in the study size before dividing by the total number of participants in all the studies:

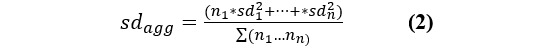



where *sd*_agg_ is the aggregated SDs, *n*_n_ is the number of participants in the study, and *sd*_n_ is the SD of the effect size of the same study.

When multiple articles reported effect sizes, the SDs were aggregated by first converting them to variances. Each variance was then weighted according to the study’s participant count and subsequently divided by the total number of participants across all the studies.

The last conversion step was to scale all correlation coefficients and SDs to the variables used in this study. This was performed by multiplying the coefficient with the quotient of the SD of the outcome variable by the SD of the predictor variable:

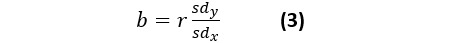



where *b* is the regression or SD, *r* is the correlation coefficient or SD, *sd*_y_ is the SD of the outcome variable *y*, and *sd*_x_ is the SD of the predictor *x*.

Using prior information and the observed data, a Bayesian structural regression was conducted using the *bsem* function of the *Blavaan* package (version 0.4-3) [26] in the R software (version 4.2.2; 2022-10-31 ucrt) through the RStudio graphical user interface (version 2023.03.0; Posit, PBC).

#### Sensitivity Analysis

To explore the impact of sampling size and different prior distributions on the Bayesian model, multiple variations of these factors were tested. The variations of the final model consisted of (1) variations in the number of adaption samples, burin samples, and samples and (2) variations in prior hyperparameters. Regarding sampling variations, the model was run with 3 variations in addition to the original model. Burin and sampling were set to the same amount in 3 steps: 5000, 10,000, and 25,000 samples. The adaption samples were in relation to these steps set to 1000, 1000, and 2500. Regarding prior hyperparameters, variations were constructed in the final model that had 5000 adaption samples, 50,000 burin samples, and 50,000 samples. They consisted of an iterative change in each intercept and slope parameter to a diffuse prior—N(0,10^5^). In addition, to investigate the more general effects of diffuse prior hyperparameters on intercepts, a model was run in which all intercepts had diffuse priors—N(0,10^5^). The results of this analysis are described in the following section.

### Ethical Considerations

The study received ethical clearance from the local research ethics committees at the intervention sites. This included the ethics committee of Città della Salute e della Scienza di Torino University Hospital and the ethics committee of San Luigi Gonzaga University Hospital, Orbassano (ethics approval reference 185/2015). The Swedish Ethical Review Authority (Etikprövningsmyndigheten; Dnr 2020-04175) granted additional approval for the analysis of pseudoanonymized data. Before the start of the study, all participants were thoroughly briefed on the study’s objectives and methods and provided informed consent by signing the necessary documentation.

## Results

### Overview

Most of the participants in the study were female, comprising 66% (66/100) of the total, and were also highly educated, with 81% (81/100) of participants reporting a college education. Patients indicated relatively good mental health scores, with a mean of 12.23 (SD 9.20) on the BDI-II, reflecting low depression levels. This was further supported by a mean score of 13.64 (SD 9.56) on the DASS-21, pointing to relatively low stress levels (Table 1).

**Table 1:** Descriptive statistics of variables in the structural equation modeling Bayesian path analysis model (N=100).

Variable	Values, mean (SD)	Values, median (range)
Depression (BDI-II^a^)	12.23 (9.20)	10 (0-43)
Stress (DASS-21^b^)	13.64 (9.56)	14 (0-38)
Use at 2 weeks (days)	5.52 (4.14)	6 (0-14)
Perceived ease of use at 4 weeks	32.5 (4.22)	33 (20-43)
Perceived ease of use at 12 weeks	32.7 (4.33)	33 (24-45)
Perceived usefulness at 12 weeks	37.1 (5.86)	38 (20-50)
Use at 12 weeks (days)	45.3 (28.14)	42 (2-100)

^a^BDI-II: Beck Depression Inventory–II.

^b^DASS-21: Depression, Anxiety, and Stress Scale–21.

The average use during the initial 2 weeks was 5.52 (SD 4.14) days. After 12 weeks, the average use was 45.3 (SD 28.14) days, showing a broad range of use volumes among participants. Participants rated the system favorably in terms of usefulness and ease of use (PEOU). The PEOU scored an average of 32.5 (SD 4.22) at 4 weeks and increased slightly to 32.7 (SD 4.33) at 12 weeks, indicating sustained positive impressions (scale maximum=45). The PU was also rated highly, with a mean score of 37.1 (SD 5.86) at 12 weeks, suggesting that the participants found the system beneficial (scale maximum=50).

### Source of Prior Information

The following section describes how previous research was used to inform some of the prior parameters included in the Bayesian SEM. The literature search yielded 1641 articles. After reviewing based on titles and abstracts, 99.21% (1628/1641) of the articles were excluded. A total of 12 articles were included for full screening. Of the 12 articles, 2 (17%) were removed owing to the qualitative nature of the method and the focus of the topics and 1 (8%) focused on the older adult population (aged ≥65 years).

A summary of all the results of the recalculations and the assumed prior distributions for all variables in the path analysis can be found in Table 2.

Table 3 provides the prior specifications used for the intercepts of outcome variables in our analyses. For each value, the table outcomes the distribution, the associated hyperparameters, and the source or bases for the selected priors.

Convergence of the prior model was assessed through divergences, trace plots, Gelman autocorrelation plots, effective sample size, and R-hat measures. During prior model testing, divergences occurred because of previous settings of the variance (disturbance) priors (ie, γ[SD] in Table 4).

As a result, the Blavaan default prior γ(1,0.5)(SD) was changed to γ(2,1)(SD) for all variances except use at 12 weeks, which was changed to γ(25,1)(SD) based on the larger variance in the range of 1 to 100. With these changes, the prior model ran without divergence. All other convergence indexes were acceptable in the prior model: (1) trace plots of all variables were horizontal, with the distribution showing even amounts of variation around the mean over samples; (2) Gelman autocorrelations were very low after the initial samples, expected because of Hamilton Monte Carlo; (3) effective sample sizes (as indicated by “neff” in *Blavaan*) ranged from 125,424 to 264,497 (mean 178,767; median 164,100); and (4) R-hat measures were all 1 within at least 4 decimal points of accuracy.

**Table 2:** Summary of priors in the model: outcome and predictor variables, distribution types, hyperparameters, and sources.

Outcome	Predictor	Distribution	Hyperparameters, mean (SD)	Prior source
Use_2 weeks_	Sex_female_	Normal	0.21 (1.23)	Abdool et al [27], Coughlin et al [28], and Kontos et al [29]
Use_2 weeks_	Education_low_	Normal	−0.34 (1.09)	Abdool et al [27], Børøsund et al [30], Coughlin et al [28], Golsteijn et al [31], and Kontos et al [29]
Use_2 weeks_	BDI-II^a^	Normal	0 (10)	Diffuse prior^b^
Use_2 weeks_	DASS-21^c^	Normal	0 (10)	Diffuse prior
PEOU^d^_4 weeks_	Use_2 weeks_	Normal	0.22 (1.12)	Abdool et al [27]
PEOU_12 weeks_	PEOU_4 weeks_	Normal	0.61 (0.46)	No previous research was identified. The prior for the correlation coefficient (*r*=0.6) was set based on the expert assessment of the authors.
PU^e^_12 weeks_	PEOU_12 weeks_	Normal	0.71 (0.18)	Abdool et al [27], Almazroi et al [32], and Dünnebeil et al [33]
Use_12 weeks_	PEOU_12 weeks_	Normal	1.42 (2.95)	Abdool et al [27]
Use_12 weeks_	PU_12 weeks_	Normal	1.02 (2.19)	Abdool et al [27]
Use_12 weeks_	Use_2 weeks_	Normal	1.36 (3.40)	Authors’ assessment (*r*=0.2)

^a^BDI-II: Beck Depression Inventory–II.

^b^Diffuse prior: noninformative prior distributions that assign broad probabilities across a wide range of parameter values, reflecting minimal prior beliefs or knowledge.

^c^DASS-21: Depression, Anxiety, and Stress Scale–21.

^d^PEOU: perceived ease of use.

^e^PU: perceived usefulness.

**Table 3:** Prior specifications for intercepts of outcome variables.

Variable	Distribution	Hyperparameters, mean (SD)	Prior source
Use_2 weeks_	Normal	0 (7)	Diffuse prior^a^ centered on 0, scale limits
PEOU^b^_4 weeks_	Normal	0 (25)	Diffuse prior centered on 0, scale limits
PEOU_12 weeks_	Normal	0 (25)	Diffuse prior centered on 0, scale limits
PU^c^_12 weeks_	Normal	0 (25)	Diffuse prior centered on 0, scale limits
Use_12 weeks_	Normal	0 (50)	Diffuse prior centered on 0, scale limits

^a^Diffuse prior: noninformative prior distributions that assign broad probabilities across a wide range of parameter values, reflecting minimal prior beliefs or knowledge.

^b^PEOU: perceived ease of use.

^c^PU: perceived usefulness.

**Table 4:** Prior specifications for error variances of outcome variables.

Variable	Distribution	Hyperparameters	Prior source
Use_2 weeks_	γ(SD)	Shape=3; scale=1	Default and prior convergence modification
PEOU^a^_4 weeks_	γ(SD)	Shape=3; scale=1	Default and prior convergence modification
PEOU_12 weeks_	γ(SD)	Shape=3; scale=1	Default and prior convergence modification
PU^b^_12 weeks_	γ(SD)	Shape=3; scale=1	Default and prior convergence modification
Use_12 weeks_	γ(SD)	Shape=25; scale=1	Default and prior convergence modification

^a^PEOU: perceived ease of use.

^b^PU: perceived usefulness.

The sensitivity analysis of prior settings showed that sampling had some effects on point estimates and the distributional range when one variable was changed to have diffuse hyperparameters; this could also be seen when all intercepts’ priors were changed to diffuse. However, regardless of these variations, no directional changes in the regression coefficients occurred (ie, from positive to negative or from negative to positive), and there were no changes that altered the interpretation of the level of uncertainty based on the 95% highest posterior density intervals. For single-variable diffusion variations, the median difference in point estimate was 1.6% (range −0.9% to 56.7%), and when all intercepts were changed to diffuse, the median difference was 0.2% (range 11.3%-37%). Changes in sampling had comparatively minor effects on the slopes, intercepts, and variances. On average, the sampling variations (n=5000|10,000|25,000) did not change the estimates at all (ie, the mean difference was 0), but the variable range changed somewhat between −3.4% and 2%.

### Posterior Fit Assessment

We computed the model fit indexes using the *gl_fits_all* function from the *Blavaan* package. This function provides Bayesian analogous structure equation model fit indexes, as suggested by Garnier-Villarreal and Jorgensen [34]. The absolute fit index, the Bayesian root mean square error of approximation, analogous to the frequentist equivalent root mean square error of approximation, was estimated to be on average 0.036 with a credible interval from 0 to 0.068. The corresponding values for the Bayesian analogs of incremental fit indexes were as follows: the comparative fit index was 0.960 (credible interval 0.893-1.0); the Tucker-Lewis index was 0.958 (credible interval 0.875-1.033); and its normalized variant, the Bentler-Bonett normed fit index, was 0.785 (credible interval 0.724-0.841). Finally, the posterior predictive *P* value was .05. These indexes are similar to their frequentist counterparts; however, they should be interpreted with caution. The aforementioned measures show a reasonable fit [35], but that only describes how well the model fits compared with very liberal null models, and it has been argued that fit indexes for Bayesian models may be less valuable for fit assessment [36].

### Bayesian Structural Regression Model Results

**Figure 4:**
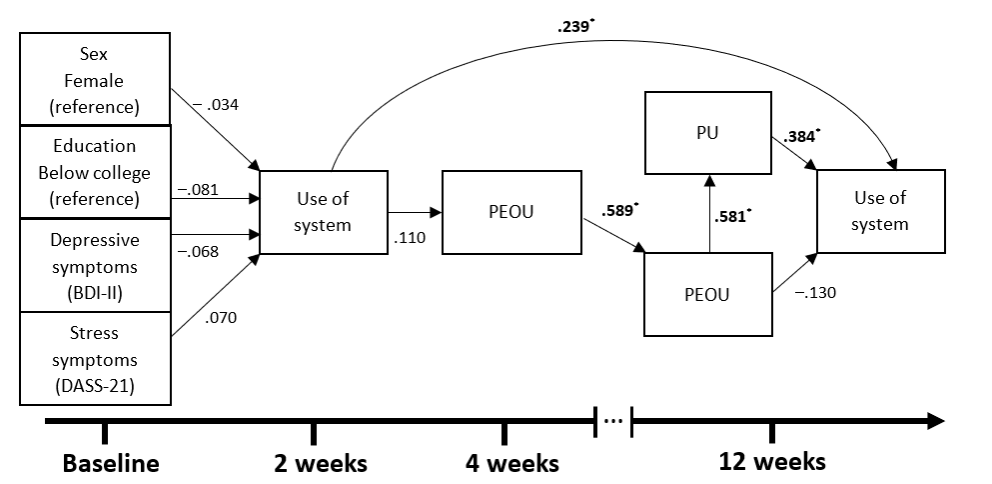
The Bayesian structural regression model results showing standardized regression coefficients (β) for all paths. *The highest posterior density credibility intervals are in the same direction (positive or negative relation), thus not crossing 0. BDI-II: Beck Depression Inventory–II; DASS-21: Depression, Anxiety, and Stress Scale–21; PEOU: perceived ease of use; PU: perceived usefulness.

The model comprised a total of 10 regression slopes, which were the main focus of this study. Of these 10 regression slopes, 4 (40%) had slopes with lower and clearer associations among variables, whereas 6 (60%) had higher uncertainty in the direction and strength of the association (Figure 4).

**Figure 5:**
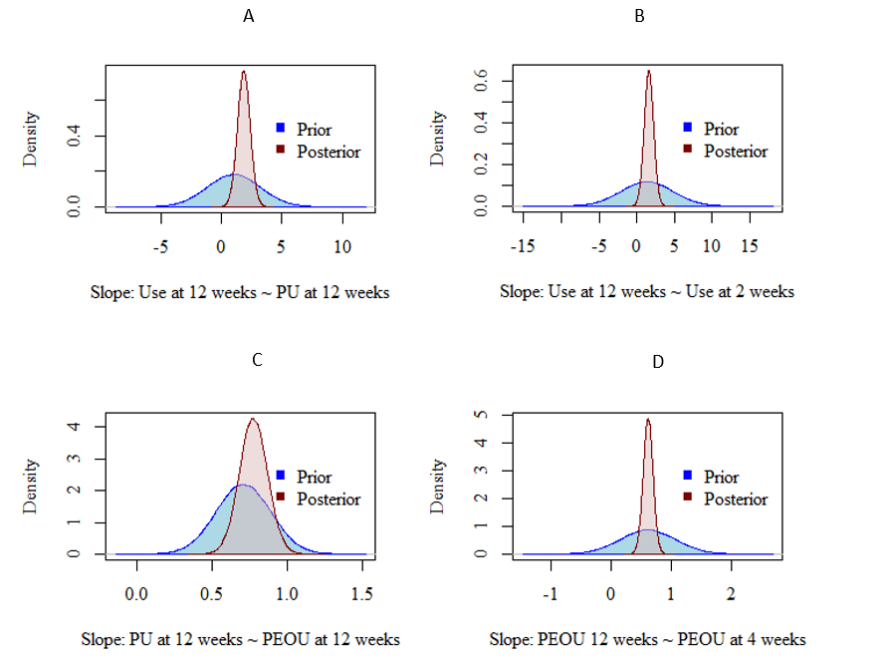
Prior and posterior distributions for the 4 associations with less uncertainty. The prior distributions are shown in blue, and the posterior distributions are shown in red. A and B show the direct predictors of use at 12 weeks. C and D show the indirect predictors of use at 12 weeks preceding the distribution in A. PEOU: perceived ease of use; PU: perceived usefulness.

Figure 4 shows that 4 regression paths, found in bold text, had clearer associations. The highest posterior density credibility intervals, noted by the asterisk, are in the same direction (positive or negative relation), thus not crossing 0. From these regressions, 2 main paths were found to predict use of the NEVERMIND system at 12 weeks. The first path is from PEOU after 4 weeks (β_PEOU 12 weeks is predicted by PEOU 4 weeks_=.589) through PU after 12 weeks (β_PU 12 weeks is predicted by PEOU 12 weeks_=.581) to the use of the system after 12 weeks (β_use 12 weeks is predicted by PU 12 weeks_=.384). The second path is the association between the use of the system after 2 weeks and the use of the system after 12 weeks (β_use 12 weeks is predicted by use 2 weeks_=.239). The prior and posterior distributions of these 4 paths are described in Figure 5. However, the third path going through PEOU at 12 weeks was unclear in its direction and strength (β_use 12 weeks is predicted by PEOU 2 weeks_=−.130).

The 6 uncertain associations had posterior coefficient distributions that contained high probabilities of both negative and positive values. The estimates for the regression coefficients, SDs, highest posterior density intervals, and standardized β coefficients are presented in Table 5. The estimates for intercept and variance can be found in Table 6.

**Table 5:** Posterior parameter estimates.

Regressions	B (SD)	HPDI^a^	β
use_2w ~ sex_female	−0.279 (0.764)	−1.765 to 1.228	−.034
use_2w ~ education_low	0.871 (0.758)	−0.624 to 2.339	−.081
use_2w ~ bdi-II^b^	−0.346 (0.743)	−1.789 to 1.125	−.068
use_2w ~ dass-21^c^	0.352 (0.702)	−1.006 to 1.754	.070
Peou^d^_4w ~ use_2w	0.112 (0.102)	−0.089 to 0.312	.110
peou_12w ~ peou_4w	0.616 (0.082)	0.457 to 0.778	.589
pu^e^_12w ~ peou_12w	0.776 (0.093)	0.590 to 0.957	.581
use_12w ~ pu_12w	1.849 (0.523)	0.830 to 2.889	.384
use_12w ~ peou_12w	−0.839 (0.691)	−2.195 to −0.520	−.130
use_12w ~ use_2w	1.604 (0.620)	0.370 to 2.805	.239

^a^HPDI: high posterior density interval.

^b^BDI-II: Beck Depression Inventory–II.

^c^DASS-21: Depression, Anxiety, and Stress Scale-21.

^d^PEOU: perceived ease of use.

^e^PU: perceived usefulness.

**Table 6:** Posterior summaries for intercepts and variances of outcome variables.

			Estimate (SD)		HPDI^a^
**Intercepts**					
	.use_2w	6.761 (2.181)		0.258 to 8.861	
	.peou^b^_4w	31.92 (0.707)		30.52 to 33.28	
	.peou_12w	13.13 (2.683)		7.434 to 18.00	
	.pu^c^_12w	11.82 (3.078)		5.666 to 17.78	
	.use_12w	14.51 (15.10)		−41.57 to 30.78	
**Variances**					
	.use_2w	17.566 (2.544)		12.85 to 22.55	
	.peou_4w	17.960 (2.574)		13.18 to 23.06	
	.peou_12w	12.384 (1.783)		9.072 to 15.94	
	.pu_12w	22.769 (3.239)		16.78 to 29.25	
	.use_12w	555.32 (70.65)		499.1 to 847.3	

^a^HPDI: high posterior density interval.

^b^PEOU: perceived ease of use.

^c^PU: perceived usefulness.

Regarding how much variance was explained by the model, the variable with the most explained variance was PEOU at 12 weeks with *r^2^* of 0.358, followed by PU at 12 weeks with *r^2^* of 0.338. The use at 2 weeks, PEOU at 4 weeks, and use at 12 weeks variables had a variance value of 0.010, 0.012, and 0.166, respectively. Thus, the model can explain some variations in attitude variables at 12 weeks, whereas use and attitude variables earlier in time were less well explained.

### Residual Covariances of Endogenous Variables

The residual covariances in the model indicate that there are some covariances that were not explained in the modeling of use at 2 and 12 weeks (Table 7). The first covariance was a positive association between female sex and system use at 12 weeks (B_use 12 weeks is predicted by female_=0.240). With regard to sex in this study, it is important to note that sex is completely confounded by type of cancer (ie, breast cancer). Therefore, the implication may be that patients with breast cancer use the system more than patients with prostate cancer. This association was planned to be modeled but dropped because of the need to limit the number of parameter assessments owing to sample size. In addition, the prior assessment of how sex is related to eHealth use is that men use eHealth more, which is the opposite association compared with the residual covariation in this case [17].

**Table 7:** Truncated residual covariance matrix of association between model variables.

Variable	Use_2 weeks_	PEOU^a^_4 weeks_	PEOU_12 weeks_	PU^b^_4 weeks_	Use_12 weeks_
Sex (female)	−0.017	−0.223	−0.245	−0.119	0.240
Education (college)	0.032	0.228	0.220	0.129	0.054
Depression symptoms (BDI-II^c^)	−0.011	0.171	0.126	0.186	−0.042
Stress symptoms (DASS-21^d^)	−0.007	0.087	−0.023	0.060	−0.057
Use_2 weeks_	N/A^e^	−0.004	0.007	0.083	0.030
PEOU_4 weeks_	N/A	N/A	−0.002	0.106	0.208
PEOU_12 weeks_	N/A	N/A	N/A	0.030	−0.119
PU_12 weeks_	N/A	N/A	N/A	N/A	0.030

^a^PEOU: perceived ease of use.

^b^PU: perceived usefulness.

^c^BDI-II: Beck Depression Inventory–II.

^d^DASS-21: Depression, Anxiety, and Stress Scale–21.

^e^N/A: not applicable.

The second and third covariances are the associations between PEOU at 4 and 12 weeks and system use at 12 weeks. However, how to interpret these residuals is unclear as the 4-week coefficient shows a positive association, whereas the 12-week coefficient shows a negative association (Table 7). Finally, these residual covariances are point estimates, and proper analysis needs to be conducted to determine the level of uncertainty of the associations.

## Discussion

### Principal Findings

The purpose of this study was to model the use of the NEVERMIND eHealth system in relation to stable baseline factors and perceptual variables following the TAM. In the 100 patients with breast and prostate cancer analyzed, the strongest predictor of use at the end of the 12-week treatment period was the PU of the system, whereas PEOU had a possible indirect influence by affecting PU. Early engagement with the system also tended to predict its use at the end of the 12-week treatment period. Although the overall model fit was deemed acceptable, the structural regressions showed a significant amount of uncertainty for baseline variables such as sex, education, and mental health symptoms related to early use.

### Interpretation of Key Findings

The PU of the NEVERMIND eHealth system at 12 weeks demonstrated the strongest association with system use at 12 weeks (β_use 12 weeks is predicted by PU 12 weeks_=.384), indicating that patients who found the system useful were more likely to use it consistently. This finding aligns with previous research on technology acceptance, which suggests that users are more inclined to adopt and continue using a system if they perceive it as beneficial for achieving desired outcomes or addressing their problems [37]. Our findings largely supported the predictions of the TAM, highlighting the PU of the NEVERMIND system as a critical determinant of its consistent use while also highlighting the need for considering additional factors such as early engagement. For instance, adding *early engagement* as an important variable in the TAM framework may provide a more comprehensive understanding of the factors influencing eHealth adoption and sustained use. In addition, the PEOU at 4 weeks exhibited a positive association with PEOU at 12 weeks (β_PEOU 12 weeks is predicted by PEOU 4 weeks_=.589), implying that patients’ initial impressions of the system’s user-friendliness persisted over time, influencing their continued engagement.

Despite these associations, the study revealed uncertainties in predicting the system’s early use based on baseline variables. Variables such as education, sex, and mental health symptoms exhibited an uncertain relationship with system use at 2 weeks, suggesting that these factors may not reliably predict early engagement with the system. Notably, there was a substantial positive residual covariance between sex (confounded by type of cancer treatment) and system use at 12 weeks (B_use 12 weeks is predicted by female_=0.240). This result suggests a potential difference in system use between patients with breast and prostate cancer, although further exploration is required owing to the confounding effect. Several explanations can be considered for the uncertainty surrounding baseline mental health symptoms’ impact on the use of the NEVERMIND system. First, the system may be well designed and effective in addressing the challenges faced by individuals with varying levels of baseline depression and stress symptoms. The personalized modules of the NEVERMIND system may have aided users in engaging with the platform irrespective of their initial symptom severity. Second, the study may have lacked sufficient statistical power owing to the low variability in baseline symptom scores among users. The duration and timing of the measurements might not have been optimal for observing the hypothesized relationship as the effects of, for example, baseline depression symptoms on use may become apparent only after a longer duration given that the treatment for depressive symptoms can take 3 to 8 months [38]. Finally, there could be other unmeasured confounding factors such as individual differences in motivation or resilience that might mask the relationship between baseline mental health symptoms and use. Our findings suggest that the influence of external user characteristics within the TAM might differ in clinical contexts, emphasizing the need for theoretical flexibility when applying the TAM in diverse settings.

The findings of this study hold valuable implications for implementing eHealth systems such as NEVERMIND. An essential insight from this study is the significance of PU in determining system use. This suggests that, when introducing eHealth technologies, caregivers must provide a thorough explanation of how the technology will enhance patients’ health, including any available evidence supporting the system’s effectiveness. By doing so, we can foster a sense of PU in patients, thereby encouraging consistent use.

In addition, our findings highlighted the influence of early system engagement on its continued use. Therefore, it would be strategic to allocate resources primarily toward monitoring, supporting, and incentivizing system use in the initial stages of an intervention. Ensuring patients’ engagement with the system early on appears more critical than maintaining these efforts throughout the entire intervention period.

### Limitations

This study has certain limitations that should be acknowledged, including the relatively small sample size, which may have limited the statistical power to detect subtle relationships. The sample was also not diverse, comprising mostly highly educated participants and a healthy population, which could restrict the generalizability of the findings to other patient populations who may have a harder time adapting to technological systems. It should also be noted that a potential limitation of our study lies in the exclusion of 8 patients who failed to complete either the usability and acceptability questionnaires or the mental health follow-up questionnaires. Although these patients did not show significant differences in sociodemographic characteristics or baseline depressive and stress symptoms, their absence could introduce a potential bias as their lack of feedback might indicate challenges with the system’s ease of use or PU.

Our approach to measuring the use of the NEVERMIND system also has certain limitations. Specifically, we considered multiple uses of the system within a single day as one instance of use because of constraints from the server-provided data for both the shirt and mobile app. This could potentially underestimate the system’s use if a person used it multiple times per day but it was recorded as a single instance. Future research may benefit from more granular tracking of use patterns, including the frequency of use per day and duration of each use, to provide a more comprehensive understanding of user engagement. However, it is also important to consider, as supported by Lally et al [14], that the quality of user engagement and the ability to derive needed support might be more critical than the sheer frequency or duration of use.

In addition, there may have been unmeasured confounders that were not accounted for in this study.

Our study also assumes that the relationships described in the Bayesian SEM hold true; however, unmeasured confounding variables may distort these relationships, leading to biased estimates. Furthermore, the uncertainty observed in some of the regression coefficients points toward potential model specification issues or inherent variability in the data that were not captured in the model. This uncertainty might pose challenges in making robust predictions about system use based on baseline variables. From a methodological perspective, the significant residual covariances observed might suggest a need to revise the model. For instance, it might be beneficial to explore whether additional variables or paths should be included in the model or whether certain relationships might be nonlinear.

The changes made to the prior model owing to divergences in the initial runs are another limitation despite carefully considering the choice of prior distributions for most of the parameters. Although these adjustments helped the model converge, they may have also influenced the resultant estimates and the interpretation of the findings.

Overall, these statistical and methodological limitations need to be acknowledged when interpreting the findings of our study and should be addressed in future research.

### Conclusions

This study offers valuable insights into the complex dynamics affecting patient engagement with eHealth systems, underscoring the importance of PU and early engagement. Therefore, it is paramount to educate patients on the system’s benefits and effectiveness to encourage early and continued use. Given the complexities of patient behavior, further research is warranted to clarify the remaining uncertainties. Addressing these gaps will pave the way for a more effective deployment of eHealth systems in patient care.

## Appendix

Multimedia Appendix 1 Perceived ease of use questionnaire.

Multimedia Appendix 2 Perceived usefulness questionnaire.

## Abbreviations

BDI-II: Beck Depression Inventory–II
DASS-21: Depression, Anxiety, and Stress Scale–21
PEOU: perceived ease of use
PU: perceived usefulness
RCT: randomized controlled trial
SEM: structural equation modeling
TAM: Technology Acceptance Model
