# Supplementary figures and images for: Predictors of the Use of a Mental Health–Focused eHealth System in Patients With Breast and Prostate Cancer: Bayesian Structural Equation Modeling Analysis of a Prospective Study
Source: JMIR Cancer. 2023 Sep 12;9:e49775. doi: 10.2196/49775 (PMC10523218; doi:10.2196/49775)

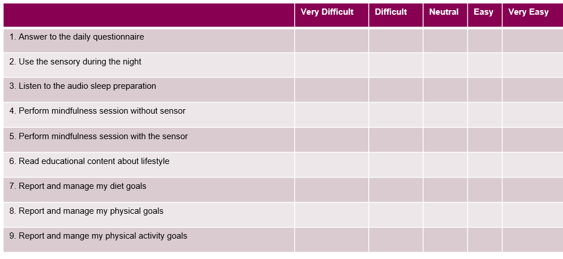

Supplement: Multimedia Appendix 1 [file cancer_v9i1e49775_app1.png]

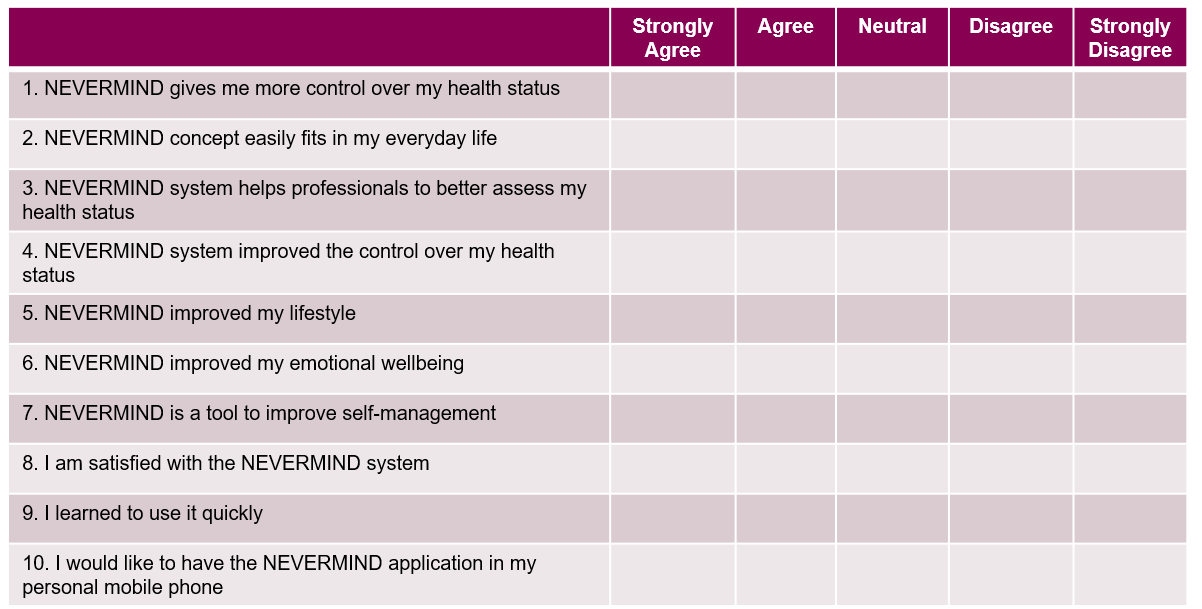

Supplement: Multimedia Appendix 2 [file cancer_v9i1e49775_app2.png]
